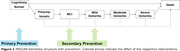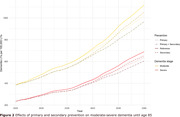# Comparing the short‐term and long‐term impact of primary and secondary prevention strategies on population‐level dementia burden: A microsimulation modelling study

**DOI:** 10.1002/alz.091332

**Published:** 2025-01-09

**Authors:** Lynn van Rosmalen, Chiara C. Brück, Frank J. Wolters, Ron Handels, Inge M.C.M. de Kok

**Affiliations:** ^1^ Department of Public Health, Erasmus MC University Medical Center, Rotterdam Netherlands; ^2^ Department of Radiology & Nuclear Medicine and Alzheimer Center, Erasmus MC, Rotterdam Netherlands; ^3^ Department of Epidemiology, Erasmus MC, Rotterdam Netherlands; ^4^ Maastricht University; Department of Psychiatry and Neuropsychology; Alzheimer Centre Limburg; School for Mental Health and Neurosciences, Maastricht Netherlands

## Abstract

**Background:**

Dementia prevention can be achieved through modification of risk factors (primary prevention) or targeted therapy against pathophysiological hallmarks of disease (e.g., amyloid‐lowering medication (secondary prevention)). To compare the effectiveness of primary and secondary prevention approaches on population dementia burden over time, we used a microsimulation approach.

**Method:**

We used the microsimulation model MISCAN‐Dementia to simulate 10 million people born between 1910‐1989, in absence and presence of dementia prevention (Figure 1). For primary prevention, we assumed it is effective if there is no brain pathology, and dementia was attributable to risk factors. We assumed that primary prevention delays the presymptomatic stage by 12 months. For secondary prevention, we assumed patients are eligible for amyloid‐lowering treatment if they are in MCI or early dementia stage and are amyloid positive. We assumed that treatment increases MCI and mild dementia stage durations by 10%. To simulate these primary and secondary interventions, we incorporated a presymptomatic dementia stage (pathology present, no cognitive decline), amyloid status, and risk factor status in the model. Both interventions are initiated in 2025. We calculated the number of life years lived with dementia per 100.000 persons <85 years by disease stage.

**Result:**

Benefits of primary prevention accrued slowly over time, from no benefit at 5 years post initiation, to around 8‐9% reduction in the number of life years with moderate‐severe dementia at 25 years after initiation. After 5 years, secondary prevention showed a 4.8% decrease in years lived with moderate dementia and 2.6% with severe dementia. This reduction increases to 5‐6% in 25 years (Figure 2). In case both interventions are applied, the effect is 0.5‐1% weaker than additive. The two modes of prevention are equally effective 19 years after initiation.

**Conclusion:**

Compared to secondary prevention, primary preventive strategies can yield a larger reduction in life years with dementia, but benefits take a much longer time to accrue. These findings underline the need for combined primary and secondary prevention initiatives and sustained, long‐term efforts to achieve the full potential of dementia prevention in the population. Sensitivity analyses to examine the robustness of the findings need to be complemented.